# In vivo PET imaging of the neuroinflammatory response in rat spinal cord injury using the TSPO tracer [^18^F]GE-180 and effect of docosahexaenoic acid

**DOI:** 10.1007/s00259-016-3391-8

**Published:** 2016-05-07

**Authors:** J. L. Tremoleda, O. Thau-Zuchman, M. Davies, J. Foster, I. Khan, K. C. Vadivelu, P. K. Yip, J. Sosabowski, W. Trigg, A. T. Michael-Titus

**Affiliations:** Centre for Trauma Sciences, The Blizard Institute, London, UK; Barts Cancer Institute, Queen Mary University London, London, UK; GE Healthcare Ltd, Amersham, UK

**Keywords:** Spinal cord injury, Neuroinflammatory respone, [^18^F]GE-180, Rat model, Docosahexaenoic acid

## Abstract

**Purpose:**

Traumatic spinal cord injury (SCI) is a devastating condition which affects millions of people worldwide causing major disability and substantial socioeconomic burden. There are currently no effective treatments. Modulating the neuroinflammatory (NI) response after SCI has evolved as a major therapeutic strategy. PET can be used to detect the upregulation of the 18-kDa translocator protein (TSPO), a hallmark of activated microglia in the CNS. We investigated whether PET imaging using the novel TSPO tracer [^18^F]GE-180 can be used as a clinically relevant biomarker for NI in a contusion SCI rat model, and we present data on the modulation of NI by the lipid docosahexaenoic acid (DHA).

**Methods:**

A total of 22 adult male Wistar rats were subjected to controlled spinal cord contusion at the T10 spinal cord level. Six non-injured and ten T10 laminectomy only (LAM) animals were used as controls. A subset of six SCI animals were treated with a single intravenous dose of 250 nmol/kg DHA (SCI-DHA group) 30 min after injury; a saline-injected group of six animals was used as an injection control. PET and CT imaging was carried out 7 days after injury using the [^18^F]GE-180 radiotracer. After imaging, the animals were killed and the spinal cord dissected out for biodistribution and autoradiography studies. In vivo data were correlated with ex vivo immunohistochemistry for TSPO.

**Results:**

In vivo dynamic PET imaging revealed an increase in tracer uptake in the spinal cord of the SCI animals compared with the non-injured and LAM animals from 35 min after injection (*P* < 0.0001; SCI vs. LAM vs. non-injured). Biodistribution and autoradiography studies confirmed the high affinity and specific [^18^F]GE-180 binding in the injured spinal cord compared with the binding in the control groups. Furthermore, they also showed decreased tracer uptake in the T10 SCI area in relation to the non-injured remainder of the spinal cord in the SCI-DHA group compared with the SCI-saline group (*P* < 0.05), supporting a NI modulatory effect of DHA. Immunohistochemistry showed a high level of TSPO expression (38 %) at the T10 injury site in SCI animals compared with that in the non-injured animals (6 %).

**Conclusion:**

[^18^F]GE-180 PET imaging can reveal areas of increased TSPO expression that can be visualized and quantified in vivo after SCI, offering a minimally invasive approach to the monitoring of NI in SCI models and providing a translatable clinical readout for the testing of new therapies.

**Electronic supplementary material:**

The online version of this article (doi:10.1007/s00259-016-3391-8) contains supplementary material, which is available to authorized users.

## Introduction

Traumatic spinal cord injury (SCI) is a devastating condition, affecting around 2.5 million people worldwide [1]. It has a substantial impact on individuals and carers, and is associated with a major socioeconomic cost [2]. There are currently no effective treatments, either in the acute or the chronic phase of SCI. Currently, the follow-up and prognosis of SCI patients is based on clinical assessments of motor and sensory function. There is a need for new approaches to assess the evolution of the condition in vivo, and in particular translational approaches which can directly link the clinic to research in experimental SCI models used for the testing new therapies. Most of the biomarkers reported in SCI patients assess structural changes associated with tissue damage, e.g. compounds such the glial protein S-100B [3] or neurofilaments [4], but do not appear to provide a sensitive prognosis for clinical use [5]. Imaging technologies can provide noninvasive tools for evaluating SCI [6, 7]. MRI is currently considered the standard approach for the prognostication of acute SCI [8] and more recently, the use of functional nuclear imaging approaches has been providing new targets for assessing the inflammatory and metabolic changes in the central nervous system (CNS), providing new perspectives on the pathophysiology of neurotrauma [9].

Traumatic SCI is associated with activation of glial cells, which is a general index of neuroinflammation (NI) [10]. The NI response after SCI is complex and multiphasic [11, 12]. The neuroplasticity-inhibiting environment driven by a persistent NI response after injury could potentially be a major therapeutic target [13]. Therefore, in vivo imaging of the NI response after SCI is a desirable target for the diagnosis and monitoring of disease progression, and also for testing therapeutic effects, in preclinical studies and in patients [14]. The most established and characterized biomarker for in vivo imaging of NI is the 18-kDa translocator protein (TSPO) [15, 16]. TSPO is a cholesterol-transport protein which is present in the outer mitochondrial membrane of cells throughout the body [17]. Upregulation of TSPO expression has been associated with glial cell activation following CNS injury and inflammation in various CNS disorders [18]. Several PET tracer ligands for TSPO, including [^11^C]-R-PK11195 [19, 20], [^11^C]PBR28 [21, 22] and [^18^F]DPA714 [23], have been investigated in both animals and humans for CNS imaging and in some studies tracers have been directly compared [23, 24]. The various tracer ligands have different characteristics in terms of sensitivity, in vivo stability and specificity of binding. Due to the potential of TSPO as a NI imaging biomarker, there is significant effort to develop better tracers and also test isotopes with longer half-lives, such as ^18^F, which may also favourably affect the logistics for PET imaging. The second-generation TSPO tracer, [^18^F]GE-180 has been shown to bind selectively with high affinity to TSPO [25].

TSPO imaging of the spinal cord (normal or injured) in small experimental animals poses anatomical and technical challenges, due to the small size of the spinal cord, its location along the vertebral column and also its proximity to many other highly metabolic organs (e.g. liver, kidneys and heart) which also show a very high uptake of TSPO tracers. It is important to investigate the signal given by a TSPO tracer in experimental SCI models in order to support its potential translation to clinical use in SCI. In this study we investigated NI after SCI using in vivo PET imaging in a rat SCI model with the novel TSPO tracer [^18^F]GE-180 which has been proven to have improved imaging characteristics with higher binding potential and lower nonspecific binding when compared to PK11195 in various models of CNS injury [26–28]. We combined imaging with ex vivo autoradiography and immunohistochemistry in order to fully characterize the potential of the ligand to detect NI in the injured spinal cord. Furthermore, to investigate its clinical value we also tested its sensitivity for monitoring the therapeutic effects of docosahexaenoic acid (DHA), an ω-3 polyunsaturated fatty acid which is known to act as a powerful neuroprotective agent in rat SCI modulating the inflammatory response associated with tissue injury [29–32].

## Materials and methods

### Animal procedures

Male adult Wistar rats weighing 265 ± 35.4 g were obtained from Charles River Laboratories (Charles River, UK). All animal procedures were approved by the Animal Welfare and Ethical Review Body at Queen Mary University of London, and the UK Home Office, in accordance with the EU Directive 2010/63/EU.

#### SCI surgery

The spinal cord was injured at vertebral thoracic level 10 (T10) in accordance with a previously established contusion model in rats [33]. Animals were anaesthetized by intraperitoneal injection of a mixture of ketamine (50 mg/kg) and medetomidine (0.2 mg/kg) in sterile saline. Following the onset of anaesthesia, the most prominent spinous process in the thoracic spine was identified and marked, as it is typically the T10 spinous process. The area was shaved and cleaned with iodine. A midline incision was performed centred on the T10 mark, and the fascia and muscle layers were pulled away from the T9 – T11 spinous processes and laminae. The T9 and T11 lateral aspects of the vertebral body spinous processes were clamped to stabilize any movement of the spinal cord. A bilateral laminectomy was performed at T10, leaving the dura exposed but intact. After securing the spinal column, a PCI3000 Precision Cortical Impactor™ (Hatteras Instruments, Cary, NC) was used with the following settings: impactor tip 2 mm, impact speed 1.5 m/s, depth 1.8 mm and dwell time 100 ms. The muscle and fascia layers were then sutured and the skin was closed. Thirty minutes after contusion, a cohort of animals received an injection via a tail vein of either vehicle (0.001 % ethanol in saline) or DHA (Sigma D2534, 250 nmol/kg), in a volume of 5 ml/kg. The DHA dose chosen was based on the findings of previous studies from our laboratory showing functional improvements in SCI rats following DHA treatment [34]. Control animals were either noninjured (naive) or subjected to a laminectomy only (LAM).

All animals were considered to have undergone a successful contusion injury as shown by a complete loss of reflex responses and hind limb movement. During the recovery phase animals had access to food and water ad libitum and were housed under a 12-h light/dark cycle. All animals received a subcutaneous injection of 0.01 mg/kg buprenorphine (Reckitt Benckiser, Slough, UK) for pre-emptive analgesia followed by postoperative dosing together with saline subcutaneously twice daily for 3 days after surgery. Bladders were manually expressed twice a day until return of bladder function (<2 ml of urine in early morning expression for three consecutive days).

For study 1 on the use of TSPO for imaging the injured spinal cord, three experimental groups were set up: SCI group (ten animals), LAM group (eight animals) and naive group (six animals). For study 2 on the use of TSPO to detect therapeutic effects, two experimental groups were set up: SCI-DHA group (six animals) and SCI-saline group (six animals). (For study design see Supplementary Fig. 1a).

### Behavioural testing

All behavioural testing and data analysis were performed blind by the experimenter. Open field locomotion assessment using the Basso, Beattie, Bresnahan (BBB) Locomotor Rating Scale [35] was carried out once on day 6 or day 7 to characterize the functional outcome after spinal contusion in the SCI groups (SCI, SCI-DHA and SCI-saline) compared with the LAM group and naive group.

### Tracer production

[^18^F]GE-180 was prepared by direct nucleophilic [^18^F] fluorination of its corresponding mesylate precursor. The preparation was performed on a GE FASTlab™ synthesizer with cartridge SPE purification and formulation [36]. Radiochemical purity was 96 ± 1 % (seven production runs) and the specific activity was 175 ± 38 GBq/μmol (seven production runs) at the end of synthesis (see Supplementary Fig. 2).

### In vivo imaging

In vivo PET and CT scans were performed using a Siemens INVEON® PET/CT scanner (Siemens Preclinical Solutions, Knoxville, TN). On day 7 after surgery, animals were anaesthetized with isoflurane (5 % induction, then reduced to 1.5 – 2 % for maintenance in 0.5 L/min air) during animal preparation and scanning. A tail vein was cannulated for tracer injection (injected dose, ID, 25.4 ± 2.4 MBq, mass dose 1.9 ± 0.1 µg/ml per animal; see Supplementary Fig. 1b, c and Fig. 2) and the animal was immediately transferred to the scanner for imaging. The animals were placed prone on a heating pad to maintain a body temperature of 37 °C. Respiration was monitored throughout the scan. Due to the equipment set-up, dosing was done outside the scanner (Inveon Acquisition Workplace 2.0) and then approximately 5 min after tracer injection, a 60-min dynamic PET scan was started (matrix 128 × 128 × 161, pixel size 0.77 mm, slice thickness 0.106 cm). Immediately after the PET acquisition, a CT scan (spatial resolution 113 μm, 80 kV, 500 μA) was performed to correct for signal attenuation and anatomical references.

### PET/CT data analysis

Emission scans were analysed as total (60 min) and dynamic (12 frames × 5 min) emission data, normalized and corrected for attenuation, scattering and radioactivity decay, and reconstructed using the OSEM 3D iterative protocol from the Inveon™ Research Workplace (IRW 4.0) software (Siemens Medical Solutions). Images were then evaluated and specific uptake was assessed by drawing circular volumes of interest in the T10 spinal cord segment, avoiding the vertebral canal and using as controls the spinal cord areas within the cervical, thoracic and lumbar areas and also in the brain and biceps muscle. Tracer uptake (%ID/g) was calculated using the volume of interest analysis using the IRW 4.0 software. For study 1, the animals imaged included SCI group, the LAM group and the naive group (data from two animals were not used due to PET/CT system failure following tracer injection), and in study 2 the animals imaged included the SCI-DHA group and the SCI-saline group.

### Biodistribution

Immediately after PET/CT imaging some of the anaesthetized rats (see Supplementary Fig. 1a) were killed with sodium phenobarbital (50 mg/kg, intraperitoneal) and the following tissue samples were collected: blood, heart, lung, salivary glands, spleen, adrenal glands, kidneys, quadriceps muscle, brain and cerebellum. The spinal cord was dissected out, separating the T9 – T11 region from the non-injured regions. Each sample was weighed and counted in a γ-counter (LKB Compugamma) along with dose standards. The percentage of ID per gram of tissue was calculated for each tissue and data were normalized for activity decay and the ID.

### Ex vivo autoradiography

In a cohort of animals (LAM group, three animals; naive group, five animals; SCI-DHA group, six animals, and SCI-saline group, six animals), the whole spinal cord was freshly dissected out from the vertebral column immediately after imaging and fully exposed to a digital autoradiography x-ray film cassette for 30 min. Films were scanned with a Cyclone phosphorimager and the region of interest (ROI) data analysed using the proprietary integral OptiQuant software. Data are expressed as digital light units (DLU) per millimetre squared, normalized to the ID ([DLU/mm^2^] × ID). Dilution standards of the tracer doses were used as reference for quantification.

### Histology

On day 7 after injury, the rats were deeply anaesthetized with sodium pentobarbital (50 mg/kg, intraperitoneal; Sagatal, Rhone Merieux, Harlow, UK) and transcardially perfused with 4 % paraformaldehyde (PFA) in 0.1 M phosphate-buffer saline (PBS), pH 7.4. Spinal cords were dissected out, postfixed in 4 % PFA for 2 h, and cryoprotected in 20 % sucrose in 0.1 M PBS at 4 °C until further processing. A 10-mm segment of the spinal cord containing the contusion injury site in the centre was dissected out and embedded in OCT medium and sectioned using a cryostat. Serial 20-μm longitudinal sections, all across the whole thickness of the spinal cord, were cut and mounted onto Superfrost Plus slides.

Randomly selected spinal cord sections from the study 1 SCI, LAM and naive animals and from the study 2 SCI-DHA and SCI-saline animals (three animals per group, three sections per animal; see Supplementary Fig. 1a) were processed for immunohistochemistry using the following primary antibodies: rabbit anti-TSPO antibody (1:1,000; Novus Biologicals, Abingdon, UK) for TSPO [37], mouse monoclonal anti-glial fibrillary acidic protein (GFAP) antibody (1:800; Millipore, Temecula, CA) for astrocytes, and goat monoclonal anti-ionized calcium binding adaptor molecule 1 (Iba1) antibody (1:800; Abcam, Cambridge, UK) for microglia. Sections were incubated in 5 % normal donkey serum for 30 min, followed by overnight incubation with primary antibodies at 4 °C in a wet chamber. Sections were washed three times with PBS before incubation for 2 h in the secondary antibodies, either donkey anti-mouse for GFAP (1:200; Alexa Fluor 568, Oregon, USA), or donkey anti-goat for Iba-1 (1:200; Alexa Fluor 594, Oregon, USA).

 Tyramide signal amplification was performed for detection of TSPO. Sections were incubated with biotinylated goat anti-rabbit antibody (1:1,000, Vector BA-1000; Vector Laboratories, Peterborough, UK). After three further washes with PBS, sections were incubated with the Vectastain® ABC reagent (avidin-biotin-peroxidase complex; 1:250 solution A and 1:250 solution B in PBS; Vector Laboratories) for 30 min at room temperature. Following three washes, sections were incubated with biotinyl tyramide (Perkin Elmer, Waltham, MA) diluted in amplification diluent (1:75) for 10 min. After a further three 5-min washes with PBS, the sections were incubated with extra-avidin FITC (1:400, E2761; Sigma) for 2 h. Finally after three washes with PBS, the sections were then counterstained with the fluorescent nuclear dye bis-benzimide (Hoechst 33342, 1 μg/ml PBS; Sigma) for 10 min, followed by a 10-min wash with PBS and then mounted and coverslipped using Vectashield fluorescent mounting medium (H-1000; Vector Laboratories, Burlingame, CA).

### Histology image capture and analysis

Image analysis and quantification were performed with the observer blinded to the group assignment. Three sections per animal were stained for TSPO, Iba-1 and/or GFAP. At least eight fields were viewed and captured per section, with four fields selected around the edge of the injury site and two fields selected rostrally and caudally to the injury site. Images were viewed at ×400 magnification and photographed using a Zeiss Axioskop 2 microscope equipped with a Hamamatsu digital camera (C4742-95), and stored using HiPic version 9.1 software. Immunoreactivity quantitative analysis was carried out using the ImageJ analysis program (ImageJ 1.48; National Institutes of Health) and using a specific ImageJ script (JVP AutoColourCellCountsRev). The specific fluorescent signals emitted under different excitation lasers were selected by thresholding and then superimposed on the thresholded nuclei area to measure cellular immunoreactivity colocalization. The level of immunoreactivity was expressed as the percentage of the area of the measuring frame that contained immunoreactivity. A Zeiss LSM 710 confocal microscope was used for further detailed histological characterization and acquisition of images (ZEN lite software; Zeiss, Cambridge, UK).

### Statistical analysis

[^18^F]GE-180 uptake values were compared using one-way ANOVA (treatment effect on the total uptake summed after the 1-h PET acquisition) or two-way ANOVA (treatment and time effect on dynamic uptake during the 1-h PET acquisition) with Tukey’s multiple comparison test to compare every mean uptake value at each time-point. BBB behaviour scores, biodistribution and autoradiography data were analysed using Student’s *t* test for paired data or one-way ANOVA for grouped data. Immunohistochemistry data quantified using ImageJ software were analysed using a one-way ANOVA followed by Tukey’s multiple comparisons. Statistical analysis was performed using GraphPad PRISM 5.0 software (GraphPad, La Jolla, CA) and differences at the 95 % confidence level (*P* < 0.05) were considered significant.

## Results

### Assessment of the functional outcome after contusion SCI

After injury, the BBB scores showed a severe locomotor impairment in SCI animals (score 6.3 ± 0.5; *P* < 0.001) while normal locomotion was observed in the LAM and the naive animals (score 21 ± 0 for both groups; Table 1). In the DHA study, treatment with DHA after injury led to a marked improvement in locomotor outcome: the SCI-DHA animals had a BBB score of 5.3 ± 0.6 compared with 1.8 ± 0.6 in the SCI-saline animals (*P* < 0.001; Table 1).

**Table 1:** BBB behaviour scores acquired once on day 6 or day 7 after injury and before PET imaging

Study	Group	No. of animals	BBB score
1 (effect of injury)	SCI	10	6.3 ± 0.5*
	LAM	8	21 ± 0
	Naive	6	21 ± 0
2 (effect of DHA)	SCI-DHA	6	5.3 ± 0.6**
	SCI-saline	6	1.8 ± 0.6

The BBB scores are presented are means ± SEM

*P < 0.05, one-way ANOVA

**P < 0.05, *t* test

### In vivo imaging

**Fig. 1:**
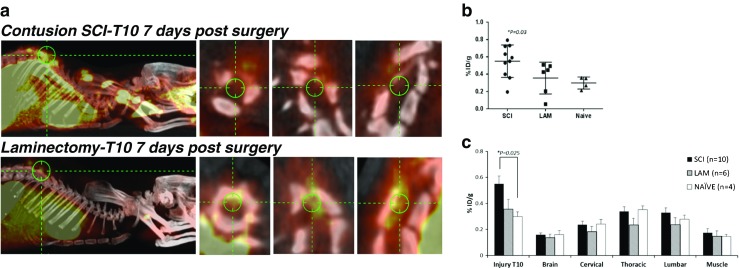
PET/CT imaging. **a** Representative PET/CT 3D MIP images in rats acquired on day 7 after SCI or laminectomy only, with higher magnification axial, coronal and sagittal 2D PET/CT images in the T10 spinal cord. Note the enhanced signal contrast in the SCI animal compared with the laminectomy only animal. **b** Quantification of the total cumulative [^18^F]GE-180 uptake in the T10 ROI during the 1-h PET acquisition shows significantly higher uptake in the SCI group (*n* = 10) than in the laminectomy group (*LAM*; *n* = 6) and naive group (*n* = 4). **c** Quantification of the total cumulative [^18^F]GE-180 uptake during the 1-h PET acquisition shows higher uptake in the T10 ROI of SCI animals than in the ROIs of other areas (cervical, thoracic excluding the T10 area and lumbar areas, brain and biceps muscle). Data are presented as means ± SEM (%ID/g); *P* < 0.05, one-way ANOVA with post-hoc analysis ()

First, cumulative [^18^F]GE-180 uptake during the 1-h PET acquisition was assessed. As shown in Fig. 1a (study 1, effect of injury), there was a significantly higher uptake in the SCI animals than in the LAM and naive animals in the ROI in the T10 region of the spinal cord (injury target) (0.55 ± 0.2 %ID/g for the SCI group vs. 0.35 ± 0.2 and 0.29 ± 0.06 %ID/g for the LAM and naive groups, respectively; *P* = 0.03; Fig. 1b). Such differences in uptake between groups in the T10 region were not detected in any other selected ROIs across the rest of the spinal cord and brain, or in the muscle (Fig. 1c).

**Fig. 2:**
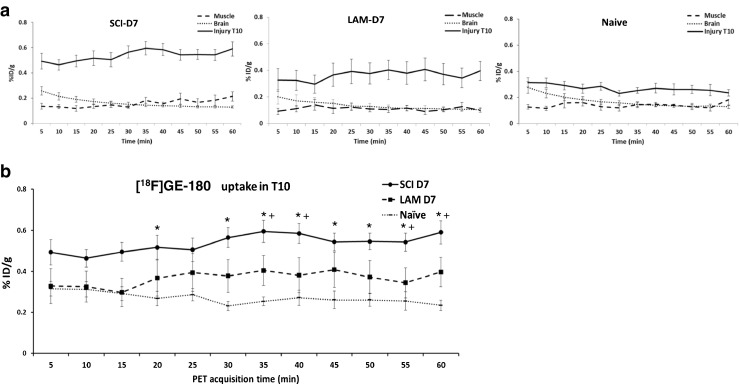
Time–activity curves of [^18^F]GE-180 uptake over the 1-h PET acquisition starting 5 min after injection (study 1). **a** Higher uptake in the T10 spinal cord ROI (*Injury T10*) compared with uptake in the brain and biceps muscle ROIs in SCI animals (*n* = 8), laminectomy (*LAM*) animals (*n* = 6) and naive animals (*n* = 4) on day 7 after surgery. **b** Higher uptake (two-way ANOVA *P* < 0.0001) in the T10 spinal cord ROI area in the SCI, LAM and naive animals; Tukey’s multiple comparison test between the groups showed a significantly higher uptake at specific acquisition times in the SCI group than in the control groups (*SCI vs. naïve, ^+^SCI vs. LAM); there were no differences in uptake between the LAM and naive groups. The data are presented as means ± SEM (%ID/g)

 To better characterize the dynamics of [^18^F]GE-180 uptake, 60-min dynamic emission scans with 5 min per frame were carried out. Scans were started 5 min after tracer injection, hence missing the initial zero condition. As shown in Fig. 2a, there was significantly higher uptake in the SCI animals than in the LAM and naive animals in the T10 spinal cord ROIs. At 30 min acquisition time (around 35 min after tracer injection), the [^18^F]GE-180 uptake in the T10 ROIs in SCI animals was significantly higher than in the naive animals (0.56 ± 0.04 %ID/g for the SCI group vs. 0.023 ± 0.02 %ID/g for the naive group at 30 min; 0.59 ± 0.05 %ID/g for the SCI group vs. 0.023 ± 0.02 %ID/g for the naive group at 60 min; *P* < 0.05; Fig. 2b). There was also significantly higher uptake in the SCI animals than in the LAM and naive animals in the time-frames 35 – 40 min and 55 – 60 min (0.59 ± 0.05 %ID/g for the SCI group vs. 0.4 ± 0.07 %ID/g for the LAM group at 35 min; 0.59 ± 0.05 %ID/g for the SCI group vs. 0.39 ± 0.07 %ID/g for the naive group at 60 min, *P* < 0.05; Fig. 2b), confirming the specific enhanced [^18^F]GE-180 uptake in the T10 ROI of the injured spinal cord. No differences in uptake were detected in the T10 ROI between the laminectomy and naive groups in any time-frame. No differences in uptake were detected in any of the groups in any time-frame in muscle (baseline measurement).

**Fig. 3:**
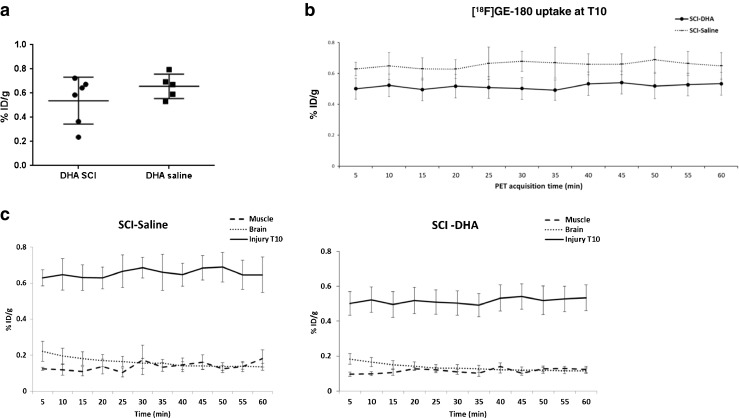
[^18^F]GE-180 uptake during PET acquisition (study 2: effect of DHA treatment). **a** Total cumulative uptake in the T10 ROI during the 1-h PET acquisition shows no significantly different uptake on day 7 between SCI animals treated with DHA (*n* = 6) and SCI animals treated with saline (*n* = 5) 30 min after injury (*t* test, *P* = 0.2). **b** Dynamic uptake in the T10 ROI in SCI animals treated with DHA and SCI animals treated with saline. No statistically significant difference was seen despite a trend towards a lower tracer uptake in DHA-treated animals (two-way ANOVA; *P* = 0.1 DHA treatment effect; *P* = 0.3 acquisition time effect). **c** Higher dynamic uptake in the T10 ROI (*Injury T10*) than in the brain and the biceps muscle ROIs over 60 min in SCI animals treated with DHA (*n* = 6) and SCI animals treated with saline (*n* = 5) on day 7 after the intervention. The data are presented as means ± SEM (%ID/g)

In study 2 assessing changes in TSPO after SCI followed 30 min later by treatment with DHA, overall both the cumulative update data and the time-frame analysis showed that the treatment induced a trend towards a decrease in tracer uptake (Fig. 3). However, the effect was not statistically significant. Thus, no significant differences in cumulative [^18^F]GE-180 uptake were seen in the T10 ROI spinal region between the SCI-DHA group and the SCI-saline group during the 1-h PET acquisition on day 7 after injury (Fig. 3a). Similarly, no differences in uptake were seen in the 60-min dynamic emission scans with 5-min frame acquisitions (Fig. 3b, c). The trend towards a decrease after DHA treatment was also detected in the other selected ROIs across the rest of the spinal cord and the brain (see Supplementary Fig. 3).

### Biodistribution and autoradiography after in vivo PET imaging

**Fig. 4:**
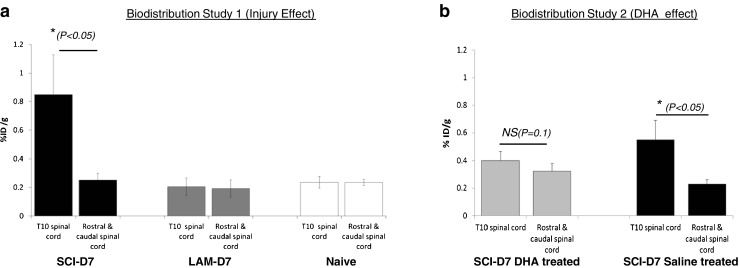
[^18^F]GE-180 in vivo biodistribution after the 1-h PET acquisition in the spinal cord (injured T10 area vs. non-injured areas rostral and caudal to the T10 region). **a** Study 1 (effect of injury): Higher uptake in the T10 area than in the non-injured areas rostral and caudal to T10 in SCI animals on day 7 after injury (*n* = 10; *t* test; *P* = 0.02). There were no differences in uptake in the spinal cord in LAM animals (*n* = 8) and naive animals (*n* = 6). **b** Study 2 (effect of DHA treatment): SCI-saline animals showed significantly higher uptake in the T10 SCI region than in the regions rostral/caudal to T10 (*P* = 0.04) on day 7 after injury, but the SCI-DHA animals (n = 6) showed no significant difference in uptake between the T10 SCI region and the regions rostral/caudal to T10 (*t* test, *P* = 0.01) on day 7 after injury. The data are presented as means ± SEM (%ID/g)

The biodistribution of [^18^F]GE-180 in the SCI animals assessed after PET imaging showed significantly higher uptake in the T10 spinal cord area tissue than in the rest of the spinal cord tissue (0.85 ± 0.3 %ID/g vs. 0.25 ± 0.04 %ID/g, respectively, *P* = 0.02; Fig. 4a). No such difference was seen in the spinal cord tissues dissected from the LAM and naive animals (0.2 ± 0.06 %ID/g vs. 0.19 ± 0.06 %ID/g, respectively, in the LAM group; 0.2 ± 0.04 %ID/g vs. 0.2 ± 0.02 %ID/g, respectively, in the naive group; Fig. 4a). The tracer biodistribution in other body tissues/organs did not show notable differences (see Supplementary Fig. 4a). Interestingly, there was lower uptake in the T10 SCI area in the SCI-DHA animals than in the SCI-saline animals, but there was no significantly different uptake between the T10 area and the other non-injured spinal cord tissue (0.4 ± 0.06 %ID/g vs. 0.3 ± 0.05 %ID/g, respectively, in the SCI-DHA group, *P* = 0.1; 0.5 ± 0.13 %ID/g vs. 0.2 ± 0.08 %ID/g, respectively, in the SCI-saline group, *P* = 0.04). The tracer biodistribution in other body tissues/organs did not show notable differences, except for higher uptake in the adrenal glands of the SCI-DHA animals (7.3 ± 1.5 %ID/g in the SCI-DHA group vs. 4.2 ± 0.8 %ID/g in the SCI-saline group, *P* = 0.01; see Supplementary Fig. 4b).

**Fig. 5:**
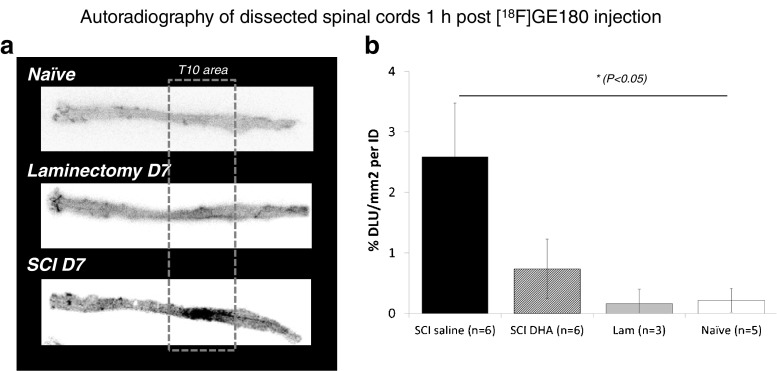
Autoradiography of dissected spinal cords 1 h after [^18^F]GE-180 PET. **a** Representative autoradiographs fully dissected spinal cords show high specific binding of [^18^F]GE-180 in the T10 area in a SCI animal 7 days after injury, in contrast to the low/absent binding in the T10 areas in naive and LAM animals. **b** Quantitative analysis of the autoradiographs shows a significant effect of SCI and DHA treatment on [^18^F]GE-180 binding in the T10 ROIs in the SCI-saline, SCI-DHA, LAM and naive animals. The data are presented as means ± SEM (one –way ANOVA, *P = 0.01)

Figure 5a shows typical autoradiography images of the dissected spinal cords after [^18^F]GE-180 CT data analysis imaging. There was preferential accumulation of radioactivity in the T10 SCI area compared with the accumulation in rest of the non-injured spinal cord 7 days after injury, and compared with the accumulation in the spinal cords of the LAM and naive animals. Quantitative analysis of the autoradiographs confirmed the significantly higher uptake of [^18^F]GE-180 in the T10 ROI of the SCI-saline animals compared with uptake in the SCI-DHA, LAM and naive animals (*P* = 0.01, one-way ANOVA with Tukey’s multiple comparisons; 2.6 ± 0.9 % DLU/mm^2^ per ID in the T10 spinal cord area in the SCI-saline animals vs. 0.2 ± 0.2 % DLU/mm^2^ per ID in the naive animals). Despite the lack of a statistically significant difference in tracer accumulation in the T10 injured spinal cord between the SCI-saline and SCI-DHA animals, an overall lower uptake was observed in the SCI-DHA animals (0.7 ± 0.5 % DLU/mm^2^ per ID in the SCI-DHA animals vs. 2.6 ± 0.9 % DLU/mm^2^ per ID in the SCI-saline animals). A similar trend in tracer uptake was also seen in the other spinal cord non-injured areas (see Supplementary Figs. 4 and 5).

### Immunohistochemistry analysis

**Fig. 6:**
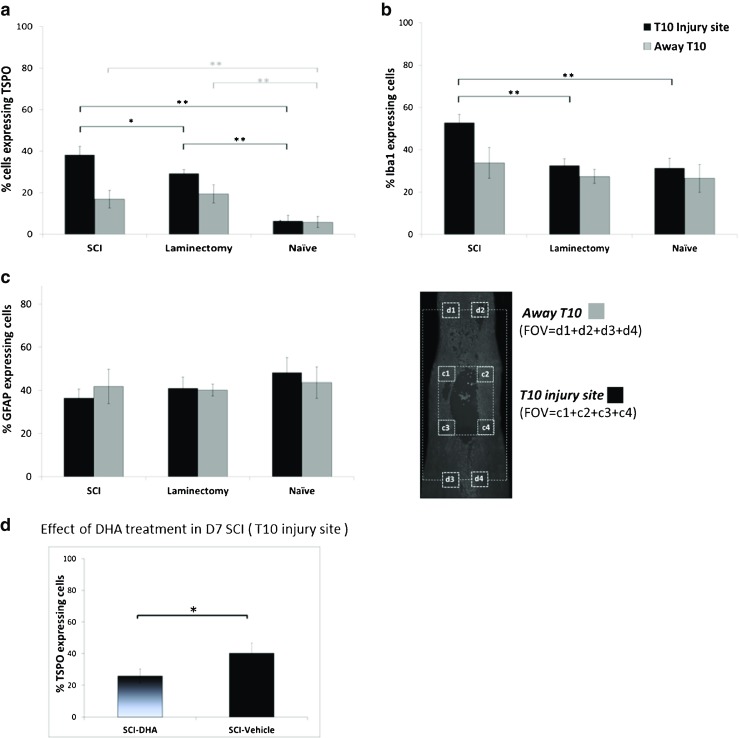
Immunohistochemical analysis of the spinal cord of SCI, LAM and naive animals (study 1) and SCI-DHA and SCI-saline animals (study 2; three animals per group) on day 7 after the intervention. The data presented are as the average percentages of cells displaying TSPO, Iba-1 or GFAP immunolabelling around the T10 injury area from fields of view (FOVs) c1 + c2 + c3 + 4, and in the areas rostral and caudal to the T10 injury site are the average percentages from FOVs d1 + d2 + d3 + d4. **a** The percentage of TSPO-expressing cells is significantly higher in the T10 injury area in the SCI animals compared with the LAM and naive animals. **b** The percentage of Iba-1-expressing cells is significantly higher in the T10 injury site in the SCI animals compared with the LAM and naive animals. **c** The percentage of GFAP-expressing cells does not differ among the three groups for either T10 injury site or the areas rostral and caudal to the injury site. **d** The percentage of TSPO-expressing cells is significantly lower in the T10 injury area in the SCI-DHA animals than in the SCI-saline animals. The data are presented as means ± SEM (one way-ANOVA with Tukey’s multiple comparisons; *P < 0.05, **P < 0.0001)

Immunohistochemistry analysis showed a significant increase in TSPO-expressing cells at the T10 injury site in the SCI animals compared with the LAM and naive animals, both around the injury site and in the areas rostral and caudal to the injury site. The percentages of TSPO-expressing cells were 38.24 ± 4.1 in the SCI animals vs. 29.29 ± 1.9 in the LAM animals (*P* < 0.05) and vs. 6.4 ± 2.7 in the naive animals (*P* < 0.0001) for the injury area, and 16.9 ± 4.1 in the SCI animals and 19.4 ± 4.3 in the LAM animals vs. 5.9 ± 2.7 in the naive animals (*P* < 0.0001) for the rostral and caudal areas (Fig. 6a). Iba-1 and GFAP immunolabelling to characterize the glial response across the injured spinal cord sections showed a significant increase in Iba-1-expressing cells (microglia) in the SCI animals compared with the LAM and naive animals at the T10 injury site (Iba-1 expressing cells 52.8 ± 3.8 % in the SCI animals vs. 32.5 ± 3.17 % in the LAM animals and 31.3 ± 4.6 % in the naive animals; *P* < 0.0001; Fig. 6b).

**Fig. 7:**
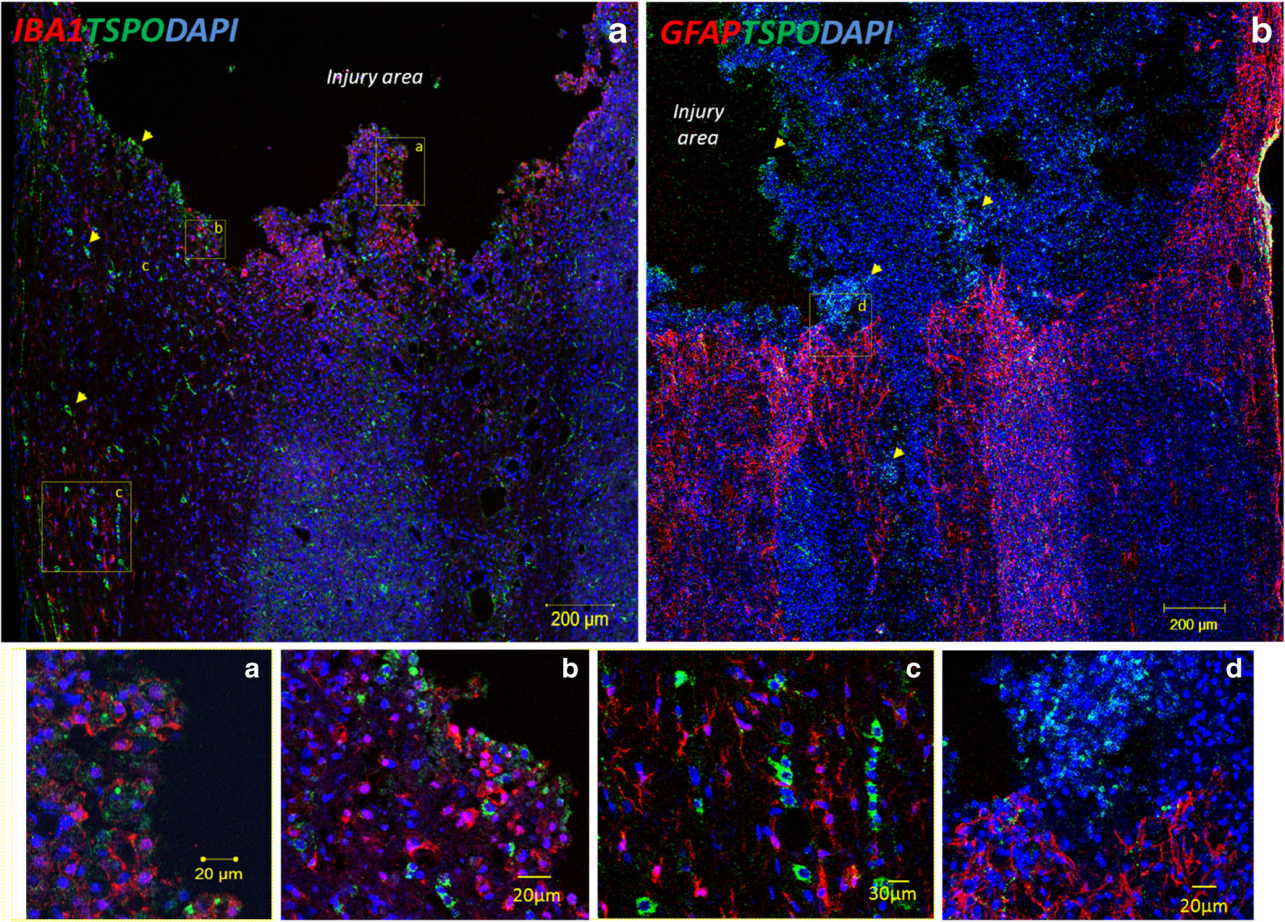
Representative confocal images of the immunohistochemical staining of spinal cord sections from SCI animals on day 7 after injury. TSPO immunolabelling can mainly be seen in the edges of the lesion (**a**, **b**), but also along the tissue extending from the injury site (*arrowheads*). **a** Microglia (Iba-1) are seen in the centre of the lesion as well as in surrounding tissue. Amoeboid and less-ramified microglia showing TSPO labelling are seen closer to the injury edges (inset *a* and *b*) while ramified microglia without or with limited TSPO labelling are seen in the tissue extending away from the lesion edges (inset *c*). **b** GFAP immunolabelling shows clear astroglial scar around the injury site. TSPO immunolabelling can mainly be seen in the centre of the lesion above the glial scar tissue (inset *d*)

There were no differences in the proportions of GFAP-expressing cells (astrocytes) between the T10 SCI area and the areas caudal and rostral to the injury site (Fig. 6c). TSPO immunolabelling was detected at the edge of the lesion site and also in the areas dorsal and caudal to the injury site in the SCI animals, but was not detected in the naive animals (Fig. 7a, b, Supplementary Fig. 6). In the SCI animals, Iba1 and GFAP immunolabelling of the glial scar showed a clear area devoid of signal at the centre of the lesion (Fig. 7a), with a dense pattern of microglia detected at the edges of the lesion core tissue with the astrocytes forming a well-defined scar border (Fig. 7b). Amoeboid and less-ramified microglia were detected at the lesion edges displaying TSPO expression (Fig. 7a, insets *a*, *b*). But away from the lesion edges, microglia showed a more ramified morphology with no colocalization of Iba1 and TSPO expression (Fig. 7a, inset *c*). However, TSPO-expressing cells were clearly detected in the regions caudal and rostral to the lesion core (Fig. 7a, arrowheads and inset *c*). TSPO immunolabelling was also apparent within the damaged core tissue above the astrocytic scar (Fig. 7, inset *d*). Tissue from SCI-DHA animals showed a significant decrease in the percentage of TSPO-expressing cells in the injury centre compared with the SCI-saline animals (25.9 ± 4.5 % in SCI-DHA animals vs. 40.19 ± 6.7 % in SCI-saline animals; *P* = 0.03).

## Discussion

This study used combined PET data analysis/CT imaging to visualize for the first time in vivo an index of the NI response in adult SCI rats and its modulation by a neuroprotective treatment. Targeting the upregulation of TSPO expression as a surrogate inflammation biomarker for CNS injury enables for the first time the selective identification of such specific NI events in an anatomically challenging injury site in a living animal model. The novel [^18^F]GE-180 tracer, with high specific binding to TSPO as shown in other CNS inflammatory models [26–28], showed differential uptake in the T10 contused SCI tissue that could be visualized and quantified in vivo, compared with the corresponding area in non-SCI animals (42 % tracer uptake increase in SCI compared with non-SCI animals). Despite the increased TSPO signal associated with other organs with high metabolism, such as the heart, kidneys and adrenal glands [15, 38] and the in situ upregulation of TSPO associated with the bone inflammatory response [34] induced by the laminectomy procedure, the PET data analysis data clearly showed higher specific uptake in the T10 SCI tissue than in the rest of the non-contused spinal cord.

 The use of combined PET data analysis/CT imaging facilitated dynamic visualization of the in vivo biodistribution and binding of the injected tracer at the injury site, in accordance with similar trends shown in other CNS injury models [26, 27], despite missing the initial activity peak due to our setting limitations for injecting the animals in the scanner. The findings were supported by the tracer accumulation detected ex vivo in dissected spinal cord after PET data analysis imaging, showing a significant increase in uptake in the T10 region compared with the rest of the non-injured spinal cord tract in SCI animals, and compared with LAM and naive animals. The in vivo differential uptake was also confirmed by the autoradiography analysis, that clearly showed the specific uptake of [^18^F]GE-180 in the injured T10 region in contrast to that seen in the LAM and naive animals. Furthermore, the upregulation of TSPO expression in the injured spinal cord was also confirmed by immunohistochemistry, that showed higher TSPO expression at the injury site in SCI animals (38 vs. 6 % in naive animals). Such increases in TSPO immunolabelling were similar to the increase in activated Iba-1-expressing amoeboid microglia at the edges of the injury site, which also showed TSPO immunolabelling. This supports the findings of previous studies in other disease models validating the use the TSPO signal in activated microglia as a biomarker of inflammation [18, 39].

 The data obtained in this study support the diagnostic value of using TSPO for in vivo imaging to monitor the ongoing NI process in the injured spinal cord, despite the small diameter (3 – 4 mm) of the injured spinal cord and the marked peripheral inflammation occurring in the nearby injured vertebrae and non-neural tissue. Previous studies have shown increased uptake of fluorodeoxyglucose (FDG) in SCI rats [40], whereas our study provides information on a specific aspect of NI occurring after CNS injury, rather than a general increase in metabolic activity. [^18^F]GE-180 PET data analysis/CT imaging was able to detect specific SCI-related NI 7 days after injury. This is a critical time for the macrophage/microglia posttraumatic inflammatory wave [41] after SCI, which in this model occurs at a time when there is ongoing hind limb locomotor impairment [31]. This minimally invasive imaging approach may allow serial monitoring of the dynamics of NI using a marker that can already be used in patients, and is thus clinically relevant [42, 43].

Another major use of TSPO imaging is to monitor the effects of therapeutic interventions developed for CNS injury. We therefore tested the use of [^18^F]GE-180 PET/CT imaging in assessing the effect of DHA treatment, which has been shown to have neuroprotective effects in CNS injury [26, 31] and stroke models [44, 45] . Studies have shown significant functional recovery following administration of a single acute intravenous dose of DHA after spinal injury in various spinal injury models [30–32]. The effects of a single injection of DHA delivered 30 min after injury are remarkably long-lasting, and interestingly, when the single bolus DHA treatment was delayed for 3 weeks or even 3 h after SCI, the functional improvement is abolished [32, 45, 46].

 Despite not being able to detect in vivo a significant increase [^18^F]GE-180 uptake in the T10 region 7 days after injury between the SCI-DHA animals and the SCI-saline animals, there was an overall trend towards a lower tracer uptake seen in the cumulative and dynamic emission PET scans (uptake was 20 % higher in SCI-saline animals than in SCI-DHA animals). This reduction in TSPO binding was better detected in the ex vivo biodistribution study, with a decrease of about 30 % in TSPO binding after DHA treatment. Interestingly, the autoradiography samples of the spinal cord from the SCI-DHA animals showed an overall decrease in TSPO binding across the whole spinal cord, including the T10 injured area, compared with the signal seen in the SCI-saline animals. This was confirmed by immunohistochemistry analysis showing lower TSPO expression in the lesion centre in the SCI-DHA animals compared with that in the SCI-saline animals (26 % in SCI-DHA animals vs. 40 % in SCI-saline animals). This significant reduction in [^18^F]GE-180 binding ex vivo supports the potential diagnostic role of TSPO imaging for monitoring NI modulatory treatments such as DHA.

 DHA exerts protective effects in microglia and neurons by maintaining, for example, the optimal size of lipid bodies, which is markedly increased during NI [47]. Smaller lipid bodies may be more mobile and effective in providing energy to mitochondria, providing a restorative effect in mitochondrial metabolism. DHA has also been linked to a reduction in oxidative phosphorylation, modulating mitochondrial respiration and reducing oxidative stress after trauma [48]. TSPO has been associated with many of these metabolic and cellular respiratory functions, supporting its modulatory role during NI, cell metabolism and cell death [49]. The mechanism involved in the NI modulatory effect of DHA and how it affects TSPO expression is not well understood and warrants further research. Our data support the NI modulatory role of DHA on day 7, yet TSPO uptake remained considerable in the SCI-DHA animals compared with the naive animals (study 1). Future longitudinal serial TSPO imaging studies on DHA-treated SCI and naive animals may help to clarify the long-term therapeutic impact of DHA treatment and its mechanisms of action.

There is scarce information on the use of TSPO imaging for monitoring therapeutic effects. Our observations support the value of the novel ligand [^18^F]GE-180 for monitoring response to treatment, as shown in a recent study which assessed treatment effects in a preclinical model of progressive multiple sclerosis [50]. Modulating glial activation and NI remains critical in neurotrauma injury, and successful modulation of NI may improve prognosis for tissue repair and regeneration [51]. The work presented here supports the value of TSPO PET imaging for assessing glial inflammatory response in vivo, and its development as a surrogate marker for NI in preclinical and clinical assessments. Despite specific limitations of preclinical PET technology (spatial resolution around 1.35 mm), functional imaging holds great potential for translational SCI research, allowing in vivo longitudinal assessment of disease progression, overcoming the cumbersome and poorly translational histopathological assessments. Furthermore, such imaging complements modalities such as MRI and CT, and provides a detailed understanding of the location and dynamics of the inflammatory response associated with spinal injury.

Imaging metabolic activity in SCI animals poses challenges, as their physiological impairment may affect radiotracer uptake. In our study, SCI animals were severely impaired (as confirmed by a BBB score of about 6.3 ± 0.5) and showed a decrease of about 20 % in body weight compared with the LAM and naive animals, which is within the range for contused SCI rat models during the first week after injury. The body weight of the animals was accounted for and normalized for tracer uptake quantification PET imaging analysis (expressed as percent injected dose per gram; quantitative analysis using SUVs showed similar differential uptake among SCI, LAM and naive animals).

Another challenge for TSPO imaging of CNS injury is associated with the lack of discrimination between the resident microglia and the infiltrated peripherally derived macrophages. Both microglia and macrophages undergo “activation” in response to neuronal injury in a variety of neurological disorders [52] and TSPO expression is highly upregulated in both cell types [18]. Experimental approaches using specifically labelled haematogenous macrophages in knock-in mouse models [53], myeloablated and bone marrow transplanted chimeric mice [54] and macrophages labelled with paramagnetic iron oxide and monitoring cell trafficking by MR imaging [55] have been used to characterize the role of systemic inflammation during CNS injury. Some of these approaches could be used to clarify the TSPO pattern of expression in resident microglia in comparison with that in infiltrating macrophages present in the injured cord. Microglia play a major role in the initial response to injury through phagocytic processing of the inflamed and injured spine, while circulatory macrophages may contribute to secondary damage and poor long-term outcome after injury [46]. From a mechanistic pathophysiological approach, it would be interesting to differentially image the TSPO expressed in microglia and those in circulating macrophages, and to clarify whether this translates into any specific clinical relevance that could affect outcomes and specific treatments. Nevertheless, our findings should open the way to develop new approaches for TSPO PET imaging as a diagnostic indicator of spinal inflammation after traumatic injury, which should help elucidate the dynamics and role of different inflammatory cells during the acute and chronic stages after injury.

### Conclusion

We report here for the first time the imaging of NI in contused spinal cords in a rat model using the novel [^18^F]GE-180 highly TSPO-specific PET radioligand. These studies should provide more insight into the preclinical and clinical relevance of assessing NI in vivo and help define the role of increased TSPO expression during CNS inflammation and repair. Overall, TSPO PET imaging may provide new approaches to improving translational research and the clinical management of SCI patients.

## Electronic supplementary material

Below is the link to the electronic supplementary material.

Supplementary figure 1 Experimental design and average body weights and average injected amounts of radioactivity per animal in each group. **a** Experimental design and table with the total number of animals used (*asterisk* Data not acquired due to PET/CT scanner failure after tracer injection, *double asterisk* Some animals were excluded due to no access to the autoradiography system on that day). **a** Data for animals in study 1 (effect of injury). **c** Data for animals the study 2 (effect of DHA). Data are presented as means ± SEM. (PPTX 220 kb)

Supplementary figure 2 Quality of the tracer from the seven production days. (PPTX 69 kb)

Supplementary figure 3 Total cumulative [^18^F]GE-180 uptake in the T10 ROI area after the 1-h PET acquisition, showing the higher uptake in the T10 ROI area in both the SCI-DHA group (*n* = 6) and the DHA-saline group (*n* = 5) on day 7 compared with the other ROI areas (cervical, thoracic excluding the T10 area and lumbar areas, brain and biceps muscle). There is no significant difference in uptake between SCI-DHA animals and the SCI-saline animals. The data presented are means ± SEM (*P* > 0.05). (PPTX 15 kb)

Supplementary figure 4 Biodistribution of [^18^F]GE-180 after the 1-h PET acquisition (PPTX 329 kb)

Supplementary figure 5 Quantitative analysis of the autoradiography of dissected spinal cords after the 1-h [^18^F]GE-180 PET acquisition in the SCI-saline animals and the SCI-DHA animals and in the LAM and naive animals. Data are presented as percent of digital light units (DLU) per square millimetre (DLU/mm^2^) for the tracer injected dose (ID). (PPTX 63 kb)

Supplementary figure 6 Representative confocal images of the immunohistochemical staining of spinal cord sections from a naive animal around the T10 region. TSPO immunolabelling is scarce and not associated with cell nuclei. Ramified microglia (Iba-1, see inset *a*) is seen throughout the whole spine, including both white and grey matter areas. (PPTX 5665 kb)
