# Supplementary figures and images for: In vivo PET imaging of the neuroinflammatory response in rat spinal cord injury using the TSPO tracer [18F]GE-180 and effect of docosahexaenoic acid
Source: Eur J Nucl Med Mol Imaging. 2016 May 7;43:1710–22. doi: 10.1007/s00259-016-3391-8 (PMC4932147; doi:10.1007/s00259-016-3391-8)

## Slide 1
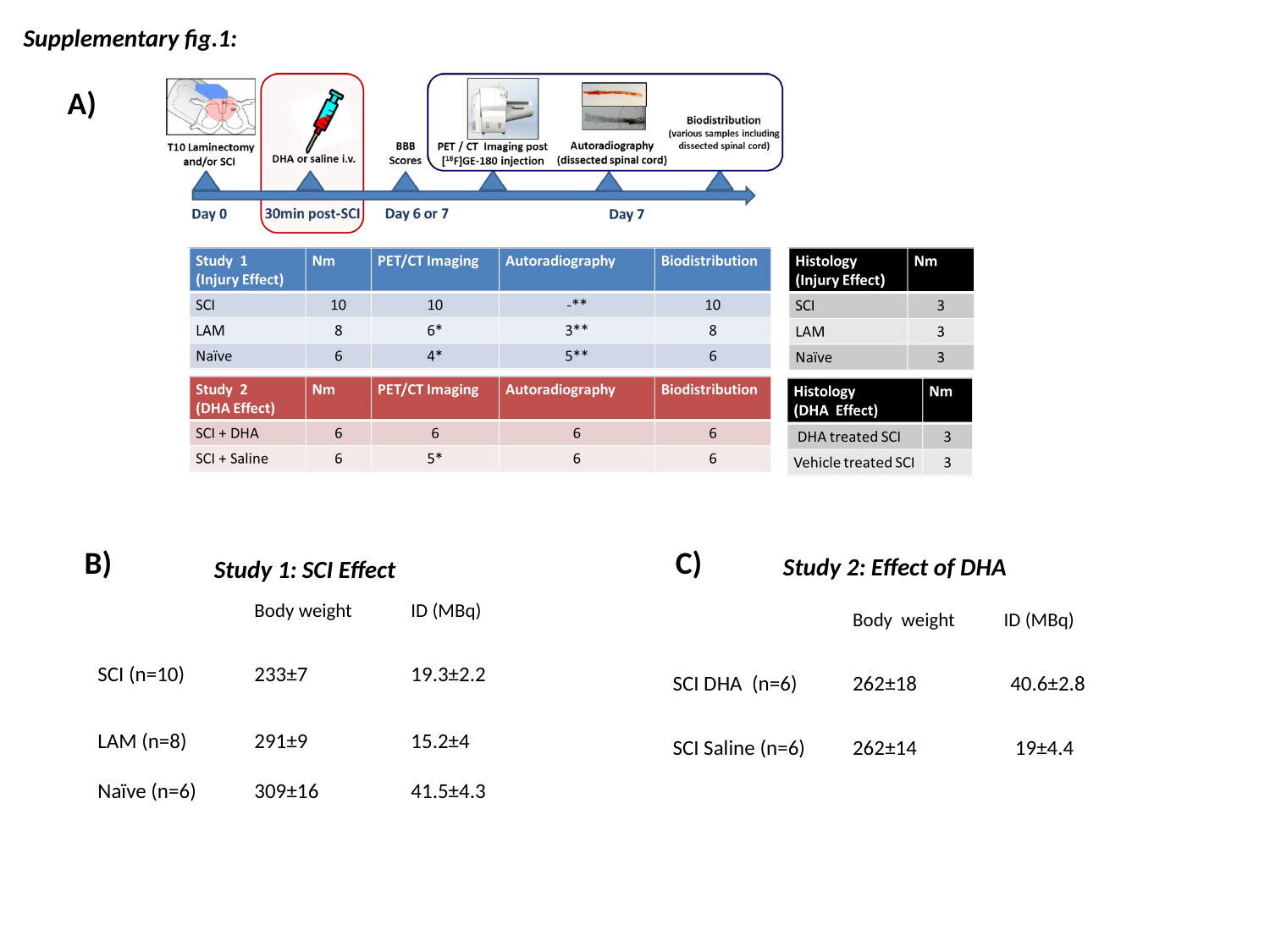

Supplement: Supplementary file 1 — Experimental design and average body weights and average injected amounts of radioactivity per animal in each group. a Experimental design and table with the total number of animals used (asterisk Data not acquired due to PET/CT scanner failure after tracer injection, double asterisk Some animals were excluded due to no access to the autoradiography system on that day). a Data for animals in study 1 (effect of injury). c Data for animals the study 2 (effect of DHA). Data are presented as means ± SEM. (PPTX 220 kb) [file 259_2016_3391_MOESM1_ESM.pptx]

## Slide 1
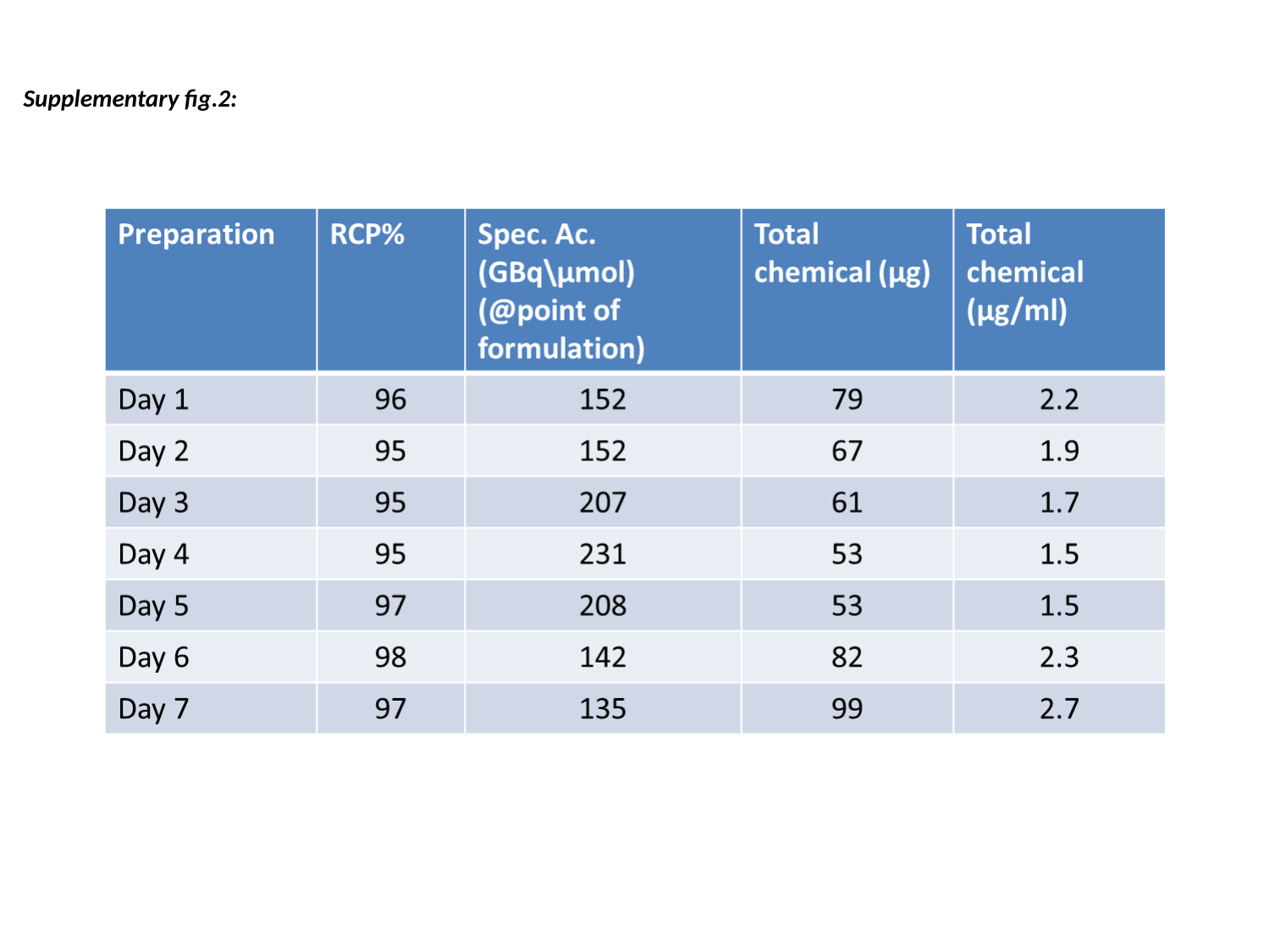

Supplement: Supplementary file 2 — Quality of the tracer from the seven production days. (PPTX 69 kb) [file 259_2016_3391_MOESM2_ESM.pptx]

## Slide 1
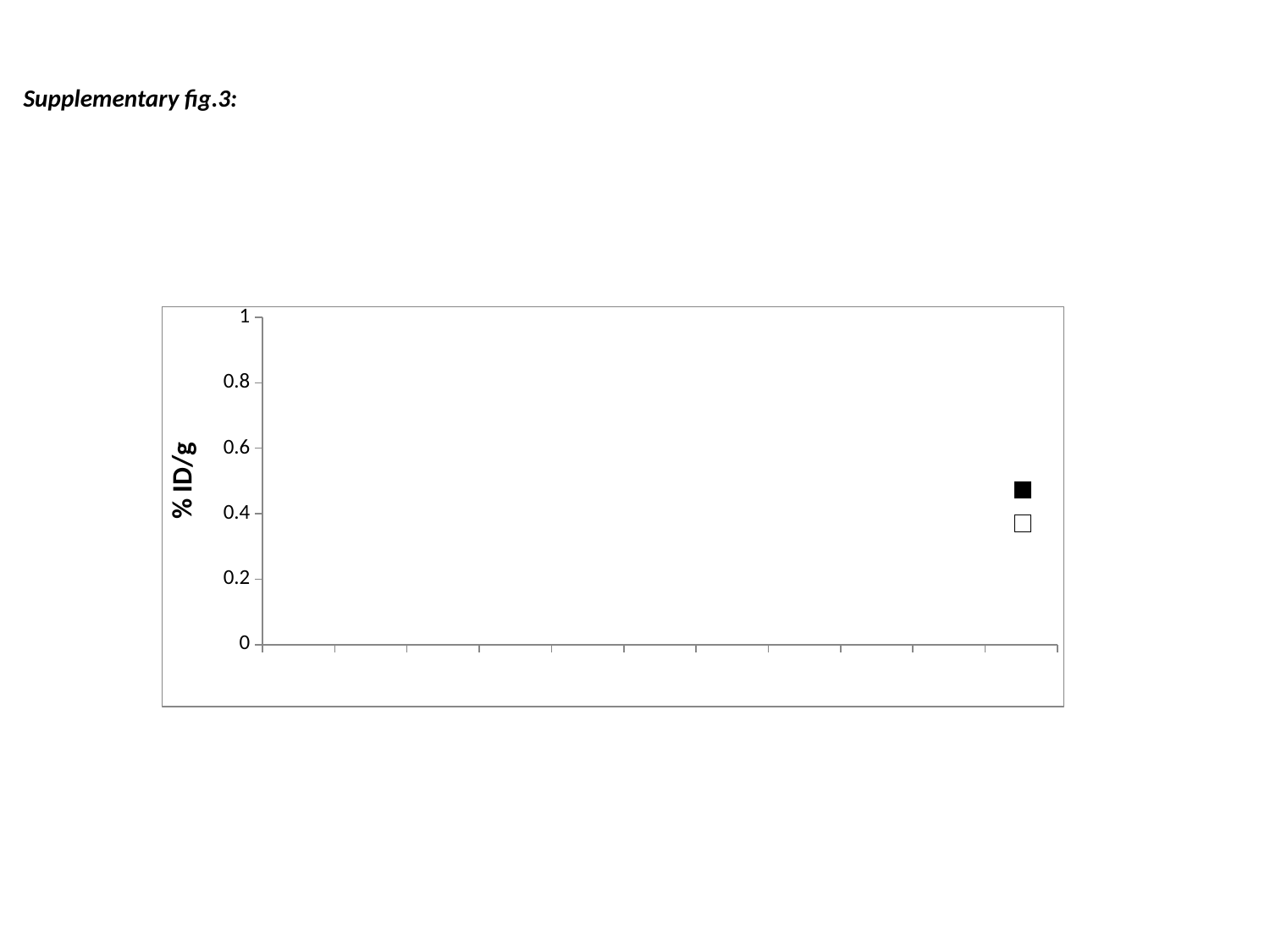

Supplement: Supplementary file 3 — Total cumulative [18F]GE-180 uptake in the T10 ROI area after the 1-h PET acquisition, showing the higher uptake in the T10 ROI area in both the SCI-DHA group (n = 6) and the DHA-saline group (n = 5) on day 7 compared with the other ROI areas (cervical, thoracic excluding the T10 area and lumbar areas, brain and biceps muscle). There is no significant difference in uptake between SCI-DHA animals and the SCI-saline animals. The data presented are means ± SEM (P > 0.05). (PPTX 15 kb) [file 259_2016_3391_MOESM3_ESM.pptx]

## Slide 1
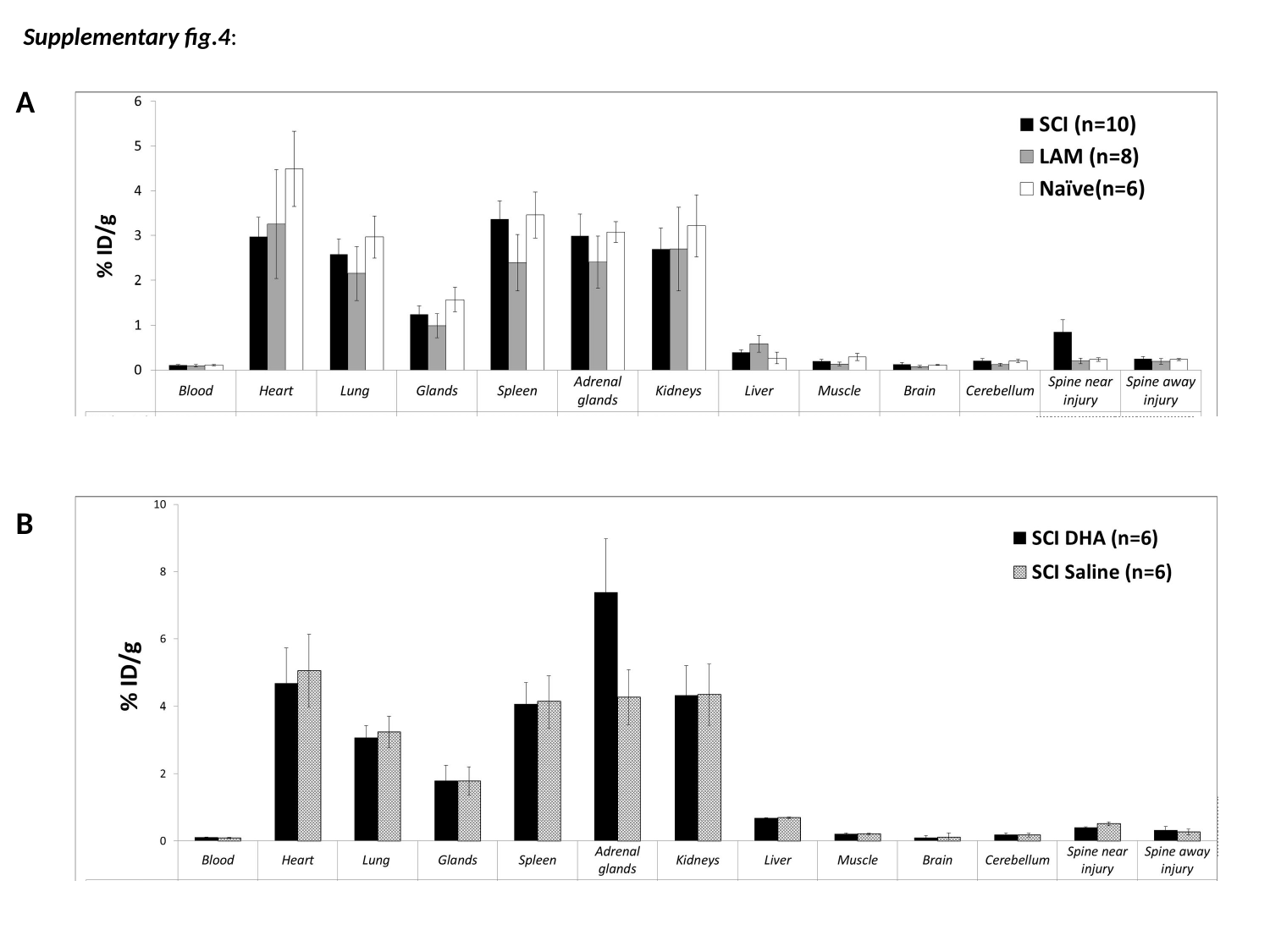

Supplement: Supplementary file 4 — Biodistribution of [18F]GE-180 after the 1-h PET acquisition (PPTX 329 kb) [file 259_2016_3391_MOESM4_ESM.pptx]

## Slide 1
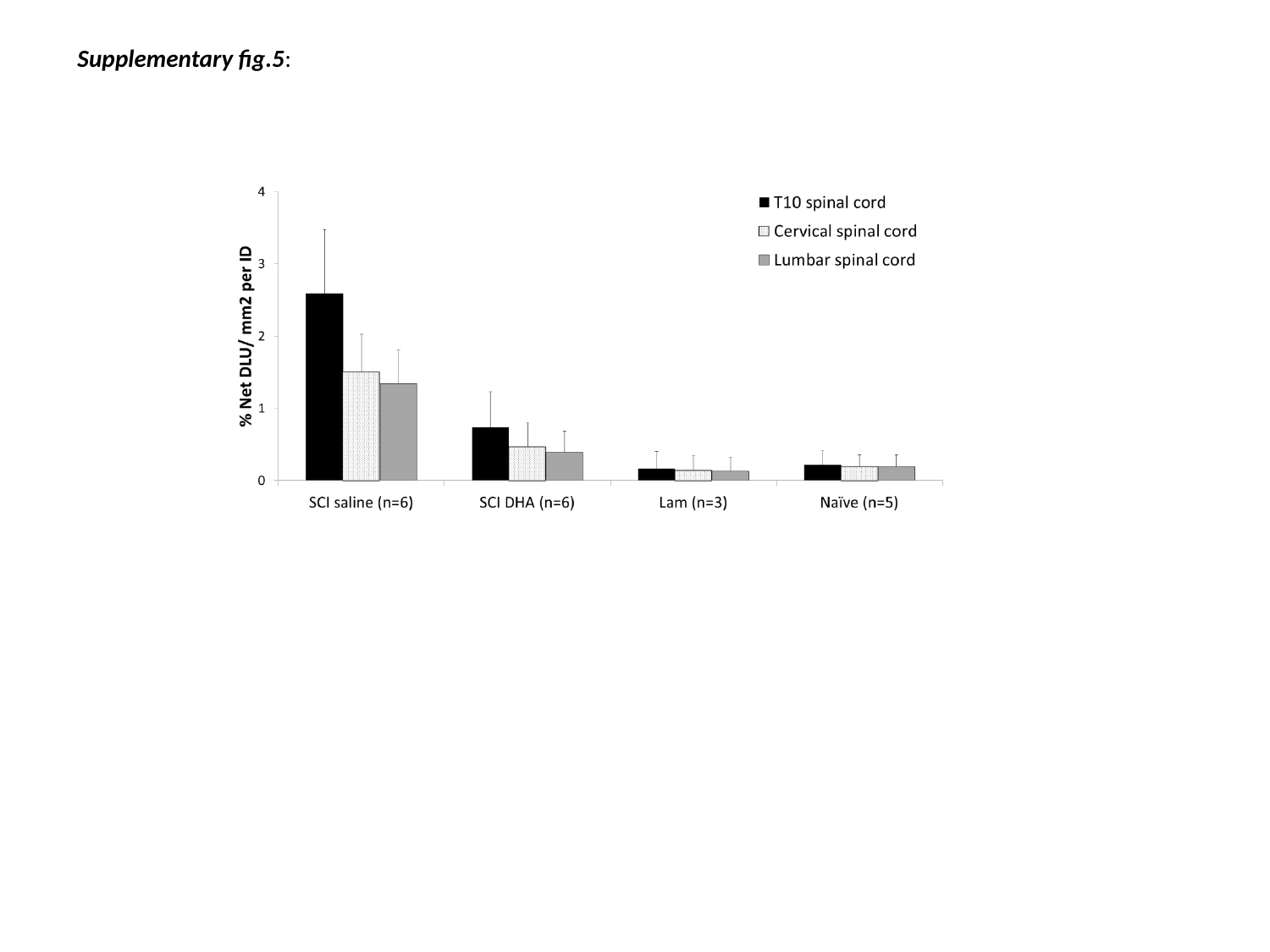

Supplement: Supplementary file 5 — Quantitative analysis of the autoradiography of dissected spinal cords after the 1-h [18F]GE-180 PET acquisition in the SCI-saline animals and the SCI-DHA animals and in the LAM and naive animals. Data are presented as percent of digital light units (DLU) per square millimetre (DLU/mm2) for the tracer injected dose (ID). (PPTX 63 kb) [file 259_2016_3391_MOESM5_ESM.pptx]

## Slide 1
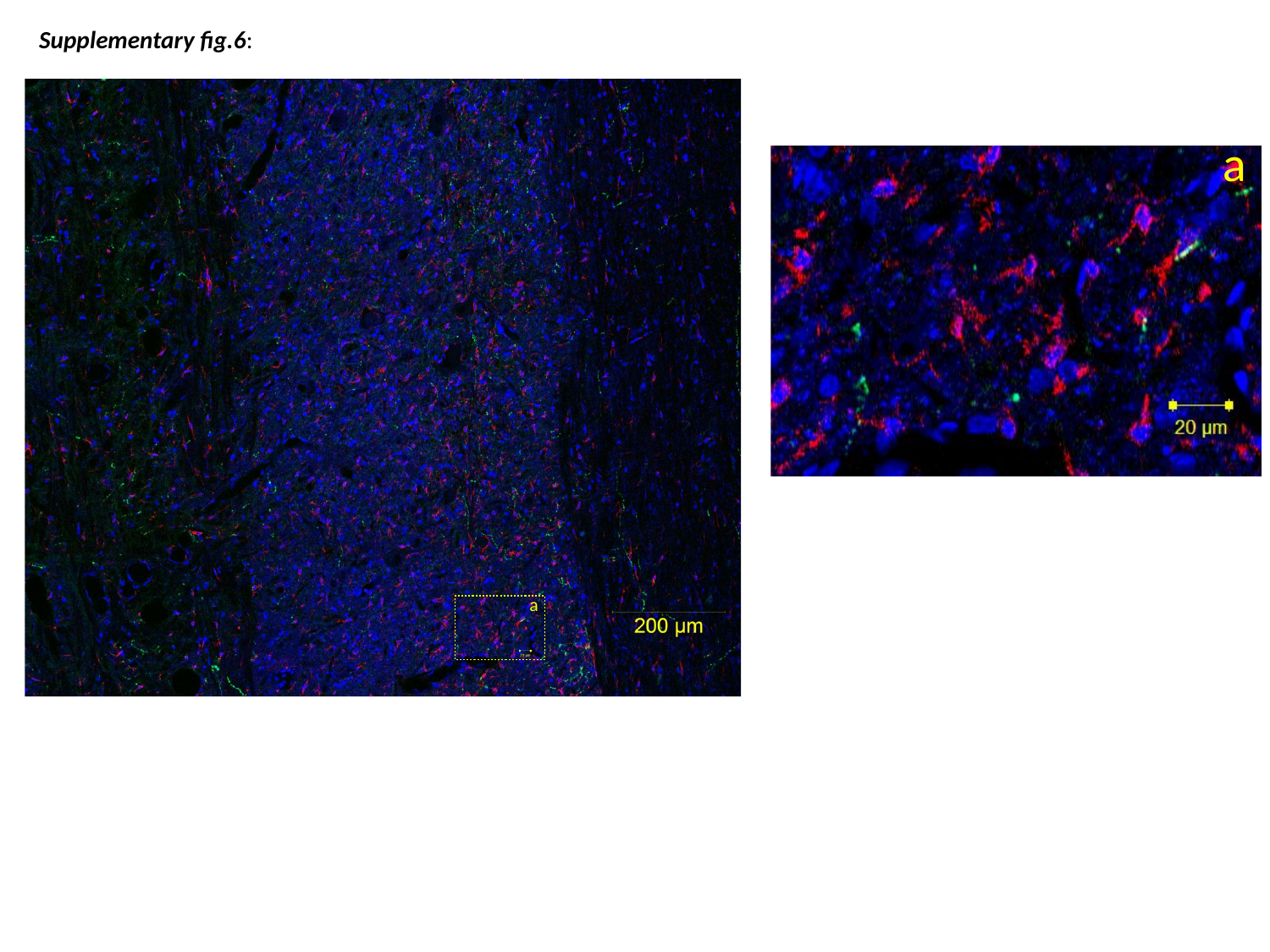

Supplement: Supplementary file 6 — Representative confocal images of the immunohistochemical staining of spinal cord sections from a naive animal around the T10 region. TSPO immunolabelling is scarce and not associated with cell nuclei. Ramified microglia (Iba-1, see inset a) is seen throughout the whole spine, including both white and grey matter areas. (PPTX 5665 kb) [file 259_2016_3391_MOESM6_ESM.pptx]
